# Skin Wound Healing Rate in Fish Depends on Species and Microbiota

**DOI:** 10.3390/ijms22157804

**Published:** 2021-07-21

**Authors:** Tery Yun, Soeun Shin, Kyungwon Bang, Mugeun Lee, Jung-Ah Cho, Myungin Baek

**Affiliations:** 1 School of Undergraduate Studies, DGIST, Daegu 42988, Korea; tery0520@dgist.ac.kr (T.Y.); gerin3@dgist.ac.kr (S.S.); bky0813@dgist.ac.kr (K.B.); mugeun0119@dgist.ac.kr (M.L.); jungahcho@dgist.ac.kr (J.-A.C.); 2 Department of Brain and Cognitive Sciences, DGIST, Daegu 42988, Korea

**Keywords:** wound healing, fish, rifampicin, skin microbiome

## Abstract

The skin is a barrier between the body and the environment that protects the integrity of the body and houses a vast microbiota. By interacting with the host immune system, the microbiota improves wound healing in mammals. However, in fish, the evidence of the role of microbiota and the type of species on wound healing is scarce. We aimed to examine the wound healing rate in various fish species and evaluate the effect of antibiotics on the wound healing process. The wound healing rate was much faster in two of the seven fish species selected based on habitat and skin types. We also demonstrated that the composition of the microbiome plays a role in the wound healing rate. After antibiotic treatment, the wound healing rate improved in one species. Through 16S rRNA sequencing, we identified microbiome correlates of varying responses on wound healing after antibiotic treatment. These findings indicate that not only the species difference but also the microbiota play a significant role in wound healing in fish.

## 1. Introduction

The skin functions as a barrier between the body and the environment. It is important to keep the skin intact to maintain the animals’ integrity. Although the basic function of the skin is very similar in most animals, its composition and organization vary between species and their habitats [1,2]. 

By using diverse model systems, the mechanisms of wound healing and strategies to improve the wound healing process have been widely studied [3,4,5]. Fish can serve as a good model to study wound healing as wound repair mechanisms in fish are very similar to those in mammals [6,7,8]. Although wound healing of diverse fish species was reportedly affected by the temperature that fish live in [9,10], the importance of host factors on wound healing in many species has not been well addressed. 

Fish skin is composed of epidermis and dermis [2,11], and wound healing mechanisms differ between wound types [6,7]. Deep wounds in fish take longer to heal than superficial and partial wounds, and recovery follows a similar process as in mammals [8]. At an initial stage, keratocytes derived from the intermediate layer of the epidermis move quickly to cover the wounded area, followed by inflammation. Neutrophils and macrophages are recruited to the wounded area to induce inflammation and activate growth factor signaling, which promotes cell proliferation and the formation of the granulation tissue. The granulation tissue forms along the wound borders and replaces the damaged tissue.

The skin houses a vast microbiota [12,13] that can migrate to the wound bed upon injury, and the role of microbiota, especially commensal bacteria, in wound healing has been studied previously [14]. Although the utility of antibiotics in wound healing has been debated [15,16], it was recently identified that commensal microbiota plays a key role in wound healing in mice. Upon skin injury, commensal bacteria move to the dermis and recruit neutrophils that activate dendritic cells (pDCs), which secrete type I IFN. Type I IFN promotes the expression of growth factors from fibroblasts and macrophages [14]. As in the mouse study, we hypothesized that the commensal microbiota on the fish skin might play a positive role in wound healing. In our preliminary results, we noticed that *Silurus microdorsalis*, which is a scaleless fish species and lives in rocky habitats, has a fast wound healing rate. Catfish, a scaleless fish with a fast wound healing rate [17], contains a large amount of mucus on their skin that assists wound healing [18]; in contrast, scales seem to delay re-epithelialization after mechanical wounds [7,8]. However, few reports compare the wound healing rates of fish with and without scales in a similar environment. To test if skin microbiota plays a positive role in the wound healing rate in fish, we screened seven fish species that encompass fish both with and without scales and are locally available and identified two fish species that showed the fastest wound healing rates. A previous study had reported that rifampicin, a broad-spectrum antibiotic, induces a drastic change in the composition of microbiota on fish skin and gut [19]. Therefore, the role of microbiota in wound healing was tested in the presence of rifampicin. Of note, we found a positive effect of antibiotics on wound healing in one species. From 16S rRNA metagenome sequencing, we could identify several bacteria correlated with differential wound healing responses to antibiotic treatment.

## 2. Results

### 2.1. Korean Bullhead and Chinese Bleak Display Faster Wound Healing Rates

**Figure 1:**
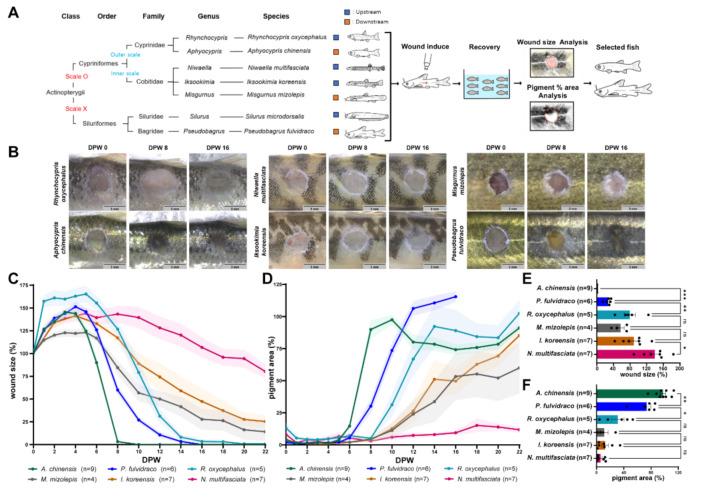
Wound healing rate in diverse fish species. (**A**) Schematic summary of the wound healing experiment. After inducing wounds to seven selected species of fish, the wound healing rate was quantified using wound size and pigmentation shown in (**B**). (**B**) Representative images of the wounds. Most individuals of *Silurus microdorsalis* died in less than 10 days after wounding (Figure S1). Scale bars: 3 mm. (**C**,**D**) Changes in wound size percent (**C**) and pigment percent (**D**). Wound size (pigment) percent: (wound size (pigment) at (n) dpw/0 dpw)*100. (**E**,**F**) Quantification of wound healing rate based on changes in wound size (**E**) and pigmentation (**F**). The wound healing rate was compared by wound size (pigment) percent at 10 dpw. Sample size (n) is denoted next to the graph. ns: not significant. * *p* ≤ 0.05, ** *p* ≤ 0.01, *** *p* ≤ 0.001.

To test the positive effect of microbiota on wound healing rates in fish, first, we tried to select fish that have fast wound healing rates. Seven locally available fish species were selected based on the differences in scales and habitats (Figure 1A). The seven fish species consisted of two fish species of the order Siluriformes (*Pseudobagrus fulvidraco*, *Silurus microdorsalis*) without scales on their skin, two fish species of the family Cyprinidae (*Aphyocypris chinensis*, *Rhynchocypris oxycephalus*) with their scales exposed to the surface of the epidermis, and three fish species of the family Cobitidae (*Misgurnus mizolepis*, *Niwaella multifasciata*, *Iksookimia koreensis*) with their scales embedded in the dermis. These fish are known to have different upstream and downstream habitats.

After wounding (3 mm in diameter; deep wound), all fish showed a similar wound healing process; wound size initially enlarged until four days post-wounding (dpw) and then contracted afterward; pigment recovery started around six dpw (Figure 1B–D and Figure S1A–C). The wound healing rate was quantified by the extent to which the wound size decreases and the pigment recovers (Figure 1E,F). Korean bullhead (*P. fulvidraco*) and Chinese bleak (*A. chinensis*) showed the fastest wound healing rates among the seven species (average wound size/pigment recovery (aW/aP) [%] at ten dpw: Korean bullhead, 25.704/77.627; Chinese bleak, 0.000/97.450). Although most *S. microdosalis* died in the middle of the experiment, they had a similar wound healing rate to that of the Korean bullhead (Figure S1, see Supplementary Materials). 

In addition to the differences between individual species, the type of scale was correlated with the wound healing rate. Wound healing was faster in two fish species of the family Cyprinidae, which have scales exposed to the surface of the epidermis, than in three fish species of the family Cobitidae, which have scales embedded in the dermis (aW/aP [%] at ten dpw: Cyprinidae, 28.299/73.806; Cobitidae, 101.503/9.759; Figure S1D,E). 

### 2.2. Rifampicin Treatment Induces Different Effects on the Wound Healing Rate

We tested if skin microbiota has a positive effect on the wound healing rate. Two fish species (Korean bullhead and Chinese bleak) were selected for testing the effect of microbiota on wound healing since they have the fastest wound healing rate. Rifampicin was used to induce changes in microbiota on fish skin. 

**Figure 2:**
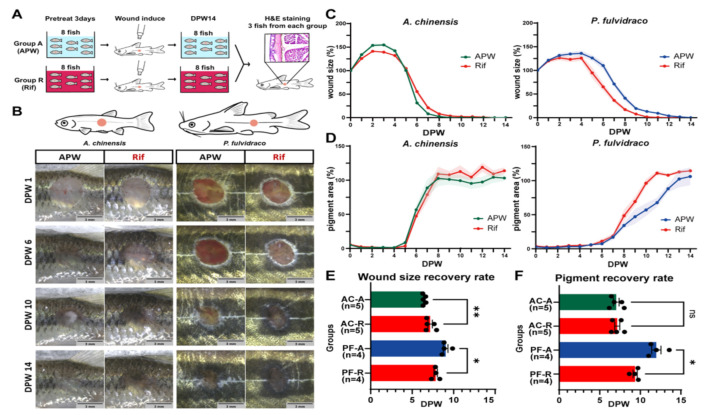
Changes in wound healing rate upon rifampicin treatment. (**A**) Schematic summary of the experiment. Fish were divided into two groups (group A and group R). Group A and group R were raised in APW and APW with rifampicin, respectively. (**B**) Representative images of wounds in group A and group R. Scale bar: 3 mm. (**C**,**D**) Wound size percent (**C**) and pigment percent (**D**). (**E**,**F**) Quantification of wound size percent (**E**) and pigment percent (**F**). The wound healing rate was compared with an average day when wound size percent becomes 20% or pigment percent becomes 80%. Sample size (n) is denoted next to the graph. ns: not significant. * *p* ≤ 0.05, ** *p* ≤ 0.01.

Before making a wound on the skin, fish were raised either in APW (group A) or APW + RIF (25 µg/mL; group R) for three days (Figure 2A) to induce changes in the microbiota. In Chinese bleaks, the initial expansion of the wound size appeared to be larger in group R than group A (Figure 2B,C), and wound size reduction occurred faster in group A than in group R (Figure 2B,C,E; average dpw of 80% wound size in group A/R, 6.448/7.170). In Korean bullheads, there was no difference in initial wound size expansion rate (1–4 dpw) between group A and group R. However, after the initial expansion, group R had a faster wound size reduction rate than group A (Figure 2B,C,E; average dpw of 80% wound size in group A/R, 9.043/7.835). While the pigmentation area of Chinese bleaks did not show any significant difference between group A and group R, pigment recovery of Korean bullheads was faster in group R than in group A (Figure 2B,D,F; average dpw of 80% pigmentation in group A/R, 11.974/9.358).

Histological analysis was performed to confirm that the wound healing of each group was completed at 16 dpw (Figure S2). Similar to the control showing clear separations of boundaries between epidermis, dermis, and red muscle, the wounded side also had distinguishable layers; however, the dermis thickened, and the skin structure was considerably disorganized.

### 2.3. Microbiomes in Two Fish Species Respond Differently to Rifampicin Treatment 

**Figure 3:**
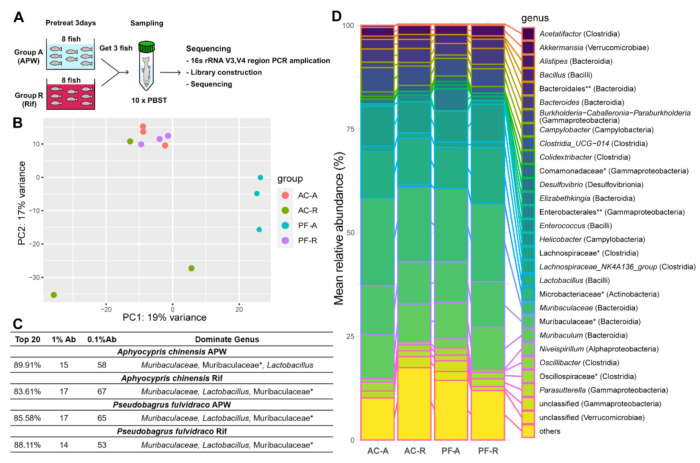
Changes in fish microbiomes upon rifampicin treatment. (**A**) Schematic summary of the experiment. (**B**) PCA plot of microbiome from *A. chinensis* and *p. fulvidraco*. *n* = 3 in each group. (**C**) Microbiome diversity of *A. chinensis* and *p. fulvidraco* in each group. All numbers are from genus-level identification. * family; Ab, abundance. (**D**) Heat map showing the top 20 dominant microbiomes in each group. Genus (class, * family, ** order).

To determine if changes in fish skin microbiota after rifampicin treatment are associated with changes in wound healing rate, metagenome analysis was performed on the mucus. Microbiomes were sampled from three fish in each group on the third day after rifampicin treatment and were analyzed using 16S rRNA metagenome sequencing (Figure 3A). The non-bacterial contamination was less than 1.8%. From the analysis of 12 samples of the metagenome, 22,459 features and 336 bacteria were identified to the genus level.

The compositions of the microbiomes of Korean bullheads and Chinese bleaks were different before the treatment, suggesting that host factors regulate the skin microbiota (Figure 3B). Rifampicin treatment changed the composition of microbiomes in both Korean bullheads and Chinese bleaks (Figure 3B). Notably, the composition of the microbiome in the rifampicin-treated Korean bullheads was similar to that in the Chinese bleaks before the treatment. Although the composition of the microbiomes changed upon rifampicin treatment, the compositions of the top twenty dominant microbiomes from each group were quite similar (Figure S3A). This result may suggest that habitats and raising environments have a particularly important effect on the major commensal microbiota on the skin. 

The percentage of the top twenty dominant genera (*family), 1% Ab, and 0.1% Ab, were very similar between the groups (Figure 3C). In all groups, the top five dominant genera or families* (class) are *Muribaculaceae* (Bacteroidia), Muribaculaceae* (Bacteroidia), *Lactobacillus* (Bacilli), *Muribaculum* (Bacteroidia), and Lachnospiraceae* (Clostridia), with differences in their ranks in each group (Figure S3A). The composition of the top five dominant genera of Chinese bleak in group A decreased by ~7.4% in group R (from 64.46% to 57.06%). In contrast, the top five dominant genera of Korean bullhead in group A increased by ~8.8% in group R (from 53.74% to 62.54% of the total microbiome). 

### 2.4. Upon Rifampicin Treatment, the Microbiome Changes with Some Correlation with the Wound Healing Rate 

**Figure 4:**
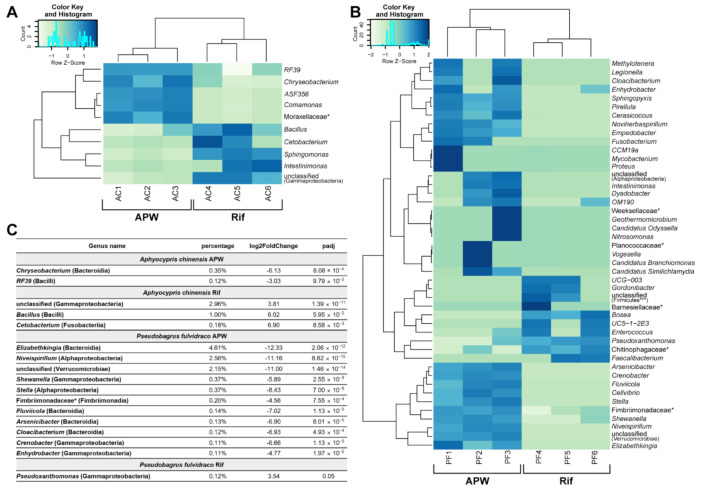
Differential abundance test of microbiome upon rifampicin treatment. (**A**,**B**) Heat maps showing taxa with a significant difference (≥ 2, FDR < 0.1) between *A. chinensis* group A and group R (**A**) and *p. fulvidraco* group A and group R (**B**). (**C**) The relative abundance of bacteria (≥ 0.1%). Genus or * family (class or *** phylum) level.

To identify correlations between changes in the wound healing rate after rifampicin treatment, we performed a differential abundance test on the microbiome of group A and group R. Bacteria showing more than a two-fold difference (FDR < 0.1) between groups were plotted at the genus level (Figure 4A–D and Figure S3B,C). Only ten genera showed differences between groups in Chinese bleak compared with 45 in Korean bullhead, which implies that the antibiotic sensitivity of bacteria in Korean bullhead is higher than that in Chinese bleak.

In Korean bullhead, bacteria with higher compositions (≥0.1%) included the genera (class) *Elizabethkingia* (bacteroidia), *Niveispirillum* (Alphaproteobacteria), and unclassified (Verrucomicrobiae) (Figure 4C and Figure S3B). In particular, the genus *Elizabethkingia* (4.61%) was 5149 times more abundant in group A than in group R. *Elizabethkingia* is commonly found in nature and is known as a bacterium that lives in fish, causing infections [18]. Another harmful bacterium that causes infections, *Shewanella* (0.37%, 59.30 fold more abundant in group A), was found in Korean bullhead. This bacterium plays a role in the decaying of fish [19]. 

In contrast to Korean bullhead group A, only ten genera were abundant in group R, and their composition was minor (≤0.12%). None of these bacteria were known to be harmful. Taken together, in Korean bullhead, the number of bacteria with higher abundance in group A than in group R was large, and many of these were found to negatively affect the fitness of the fish. In contrast, the number of bacteria with higher abundance in group R than group A was relatively small, and these were not known to be harmful. The results suggest that the difference in wound healing rate between Korean bullhead in group A and group R could be attributed to the harmful bacteria in group A, which were reduced in composition upon rifampicin treatment.

In contrast to Korean bullhead, Chinese bleak showed only a small change in the composition of the microbiome upon rifampicin treatment (Figure 4C and Figure S3B). The genera (class) *Chryseobacterium* (Bacteroidia) and *RF39* (Bacilli) were abundant in group A (genus/abundance/folds: *Chryseobacterium*/0.35%/70; *RF39*/0.12%/8.17). In group R, the genera (class) unclassified (Gammaproteobacteria) and *Bacillus* (Bacilli) were abundant (genus/abundance/folds: unclassified (Gammaproteobacteria)/2.96%/14.03; Bacillus/1.00%/64.89).

## 3. Discussion

In this study, we identified differences in the wound healing rate between seven species that were raised in the same environment. Upon wounding, no general difference was observed between fish with and without scales. However, the wound healing rates differed significantly depending on the type of scales; the wound healing rate was faster in fish with exposed scales than in those with deeply embedded scales. We have no explanation for the actual mechanisms of how these two types of scales influence wound healing. Differences in the composition of skin between fish species may underlie the varied wound healing rate. The presence of club cells varies between fish species, and club cells were presumed to perform protective functions against external stressors, including mechanical wounds [20,21]. However, the fish with their scales embedded in the skin were also reported to have club cells on their skin [22,23], implying that simply the presence of the club cells could not support the difference in wound healing rates. Other factors, including the difference in the density of club cells, the response of club cells upon mechanical wounding, and the contents in the club cells, would play a role in varied wound healing rates [20]. Confirming the role of club cells in varied wound healing rates requires further research.

Among seven local fish species, we identified two fish species (Korean bullhead and Chinese bleak) that showed the fastest wound healing rates. Since the seven fish were raised in similar environments, the differences in wound healing rate could be attributed to host factors, including genetic factors, the migration and functions of keratocytes and immune cells, and cell proliferation [6,7,8]. In addition, the commensal microbiota can affect wound healing mediated by type I IFN-dependent and IL-1β repair mechanisms [14,24]. However, in contrast to the recent reports, we identified a negative role of microbiota in wound healing of fish skin. Upon rifampicin treatment, only Korean bullhead showed a positive response to wound healing. The difference between the mouse study and our study could be attributed to the different environments in which the animals were raised. In the mouse study, mice were kept in a specific pathogen-free or relatively clean environment; therefore, after wounding, few environmental microbiotas may have had access to the wound bed [25]. In contrast, the fish in our experiment were exposed to water that could be a source of very diverse microbiota [17,26]. Moreover, from 16S rRNA metagenome sequencing, we found several harmful bacteria on the skin of Korean bullhead. Upon rifampicin treatment, the composition of those bacteria was severely reduced. Since the fish were raised in the same condition and showed no signs of infection, we infer that any difference caused by rifampicin treatment may have resulted from changes in the composition of commensal microbiota and not from infection by pathogens. Further research is needed to evaluate the effects of removing harmful bacteria that we found on healing wounds.

Furthermore, we could not entirely rule out the possibility of some differences in the mechanisms of the wound healing process mediated by microbiota; therefore, it would be interesting to examine whether upstream and downstream signaling pathways of type I IFN-dependent and IL-1β-dependent repair mechanisms are conserved or divergent between mammals and fish upon antibiotic treatment. 

In summary, we have shown that wound healing rates vary between fish species and that the composition of microbiota had a correlation with wound healing rate. This suggests that there would be some host factors speeding up the wound healing process and that the presence of specific microbiota can facilitate or inhibit the wound healing rate. A large number of the recent microbiome and genome analyses performed on diverse vertebrate species should be harnessed to identify the host factors and the molecules mediating the interactions between hosts and microbiota during wound healing. Our study in fish, where the presence of host factors controlling wound healing rates and microbiome correlates of improved wounding healing rates were presented, offers a valuable resource for further research that focuses on improving the wound healing process in vertebrates including both fish and humans.

## 4. Materials and Methods

**Fish culture.** Seven local fish species (*A. chinensis*, *M. mizolepis*, *N. multifasciata*, *P. fulvidraco*, *R. oxycephalus*, *S. microdorsalis*, and *I. koreensis*) were obtained from commercial aquariums in Seoul and Gwangju, Republic of Korea. Fish at their adult stage were selected based on their size [27,28,29,30,31]. Each fish species was raised in standard laboratory conditions at 25 °C in 12 h light/12 h dark cycles in 20 L of autoclaved artificial pond water (APW: 0.33 g/L CaCl_2_, 0.33 g/L MgSO_4_, and 0.12 g/L NaHCO_3_ in deionized water from Milli-Q system, Darmstadt, Germany). Fish were fed daily with flake food (TetraFin; Tetra, Blacksburg, VA, USA; 5 mg/fish). All experiments on fish were conducted under the approval of the IACUC at DGIST (Approved at 20/07/2012; Approval No. DGIST-IACUC-20040202-0000).

**Wound generation and antibiotic treatment.** To introduce the wound, all fish were anesthetized using MS-222 solution (Sigma-Aldrich, St. Louis, MO, USA; 0.17 g/L APW). Wounds were introduced onto the skin with a reusable rapid punch kit (WPI, Sarasota, FL, USA; 3.0 mm tip). For *A. chinensis* and *R. oxycephalus*, wounds were introduced onto the left flank directly anterior to the anal and dorsal fins; for other fish, wounds were introduced at the point where the anterior and the posterior were divided in a 2:1 ratio considering the shape of the body. Rifampicin solution (KisanBio, Seoul, Korea; 25 μg/mL APW) was used, assuming that the steady-state internal concentration in fish becomes similar to the concentration in water [19]. The APW and APW with rifampicin (APW + RIF) in the aquarium were changed once a week to maintain antibiotic effectiveness.

**Imaging and quantification of wounds.** To track the wound healing rate, each fish was anesthetized using MS-222 solution, and the images were taken using a digital camera connected to a stereo-microscope (SMZ 745T; Nikon, Tokyo, Japan). Wounds were imaged every day until six days post-wounding (dpw) for all fish species, followed by imaging every two days. In the experiment with rifampicin, wounds were imaged every day. In both experiments, seven fish from each species were imaged. Wound size and pigment were quantified using Image J (ver.1.53c). Each wound was characterized based on the color difference between the wounded and unwounded area. The wound size was estimated by identifying the edges of the wound and measuring the area.

**Microbiome sampling.** Two species, *P. fulvidraco* and *A. chinensis* were divided into two groups of eight. One group was raised in APW and the other in APW + RIF. The microbiota were harvested according to methods described previously [19]. Briefly, the mucus of each fish was extracted by adding PBST (137 mM NaCl, 10 mM phosphate, and 0.1% Tween 20, pH 7.4) in a sterile 15 mL conical tube and then vortexed for 2 min, pausing every 20 s. The mucus extracted from the PBST solution was centrifuged for 2 min at 13,000 rpm at room temperature to remove the top layer. 

**16S rRNA metagenome sequencing.** Total genomic DNA extraction was performed using a QIAamp DNA Stool Mini Kit (Qiagen, Germantown, MD, USA). The concentration of DNA was measured using a Qubit 3.0 Fluorometer (ThermoFisher, Waltham, MA, USA) to ensure that adequate amounts of high-quality genomic DNA had been extracted (>1 ng/μL). The V3-V4 hypervariable region of the bacterial 16S rRNA gene was amplified using PCR. PCR was performed using two primers: the forward primer (341F: 5′-TCGTCGGCAGCGTCAGATGTGTATAAGAGACAGCCTACGGGNGGCWGCAG-3′) and reverse primer (806R: 5′-GTCTCGTGGGCTCGGAGATGTGTATAAGAGACAGGACTACHVGGGTATCTAATCC-3′). A total of 5 ng DNA was used for doing PCR. The reaction was set up as follows: extracted genomic DNA 2.5 μL; amplicon PCR forward primer (1 μM) 5 μL; amplicon PCR reverse primer (1 μM) 5 μL; 2× KAPA HiFi Hot Start Ready Mix 12.5 μL (total 25 μL; Roche, Wilmington, MA, USA). PCR was performed in a T100 Thermal Cycler (Bio-Rad, Hercules, CA, USA) using the following program: 1 cycle of denaturing at 95 °C for 3 min, followed by 25 cycles of denaturing at 95 °C for 30 s, annealing at 55 °C for 30 s, elongation at 72 °C for 30 s, and a final extension at 72 °C for 5 min. Under this PCR condition, the non-template reaction produced no PCR products.

AMPure XP beads (A63881; Beckman coulter, Brea, CA, USA) were used to purify the free primers and primer dimer species in the amplicon products. To sequence, the amplicon, dual indices, and Illumina sequencing adapters were attached using the Nextera XT Index Kit (FC-131-2001; Illumina, San Diego, CA, USA), and the amplicon was purified again using AMPure XP beads. Before sequencing, the DNA concentration of each PCR product was determined using a Qubit 3.0 Fluorometer, and the quality of the amplicon was tested using a bioanalyzer (2100 Bioanalyzer; Agilent, Santa Clara, CA, USA). The amplicons from each reaction mixture were pooled in equimolar ratios based on their concentration. The sequencing was performed by Sanigen Inc. (Suwon, Korea) using the Illumina MiSeq system. The paired-end MiSeq Illumina reads (2 × 300 bp) were processed using QIIME2 (version 2020.8). Artificial sequences were removed using Trimmomatic (version 0.38.jar). Denoising was performed using dada2 in QIIME2. A 16S rRNA database called Silva and machine learning techniques were used to classify the flora. Furthermore, 16S rRNA from chloroplasts and mitochondria were additionally removed.

**Statistical analysis.** The statistical analysis of sequencing data was performed using R studio (ver.4.0.4). Read counts were normalized using DESeq2 (ver.1.30.1). After the normalization, a PCA plot was generated using the plotPCA. Using the ggplot2 (ver.3.3.3), the top twenty dominant bacterial species present in each group were described as bar plots of taxonomic classification. Heatmaps were produced using Heatmap.2 (gplots ver.3.1.1). The statistical analysis of wound healing rate was performed using the GraphPad program (ver.9.1.0). Group comparisons were performed using the Mann-Whitney t-test, and statistical significance was set at *p*-value < 0.05.

**Histological analysis.** Hematoxylin and eosin (H&E) staining was performed as follows: after anesthetizing the fish in MS-222, *P. fulvidraco* was perfused with 10 mL 1× PBS for 1 min, followed by perfusion with 10 ml 4% PFA for 1 min. For *A. chinensis*, the tissue was quickly harvested without perfusion. The skin tissue of the fish was cut and postfixed using 4% PFA overnight at 4 °C. After fixation, the skin was embedded in OCT (Sakura, Torrance, CA, USA) and cryosectioned with a thickness of 30 μm using a cryostat (CM3050S; Leica, Wetzlar, Germany). H&E staining kit (Vector Lab, Burlingame, CA, USA) was used for the staining. The stained tissues were imaged using an optical microscope (DM500; Leica, Wetzlar, Germany) with a digital camera (ICC50E; Leica, Wetzlar, Germany).

## Data Availability

The data presented in this study are available upon request.

## Supplementary Materials

The following are available online at https://www.mdpi.com/article/10.3390/ijms22157804/s1, Figure S1: Wound healing rate of diverse fish species, Figure S2: Skin organization after wound healing, Figure S3: Microbiome abundance in each group.
