# Supplementary material for: Skin Wound Healing Rate in Fish Depends on Species and Microbiota
Source: Int J Mol Sci. 2021 Jul 21;22(15):7804. doi: 10.3390/ijms22157804 (PMC8346108; doi:10.3390/ijms22157804)
Supplement: Supplementary file 1 [file ijms-22-07804-s001.zip › ijms-1273448-supplementary.pdf]

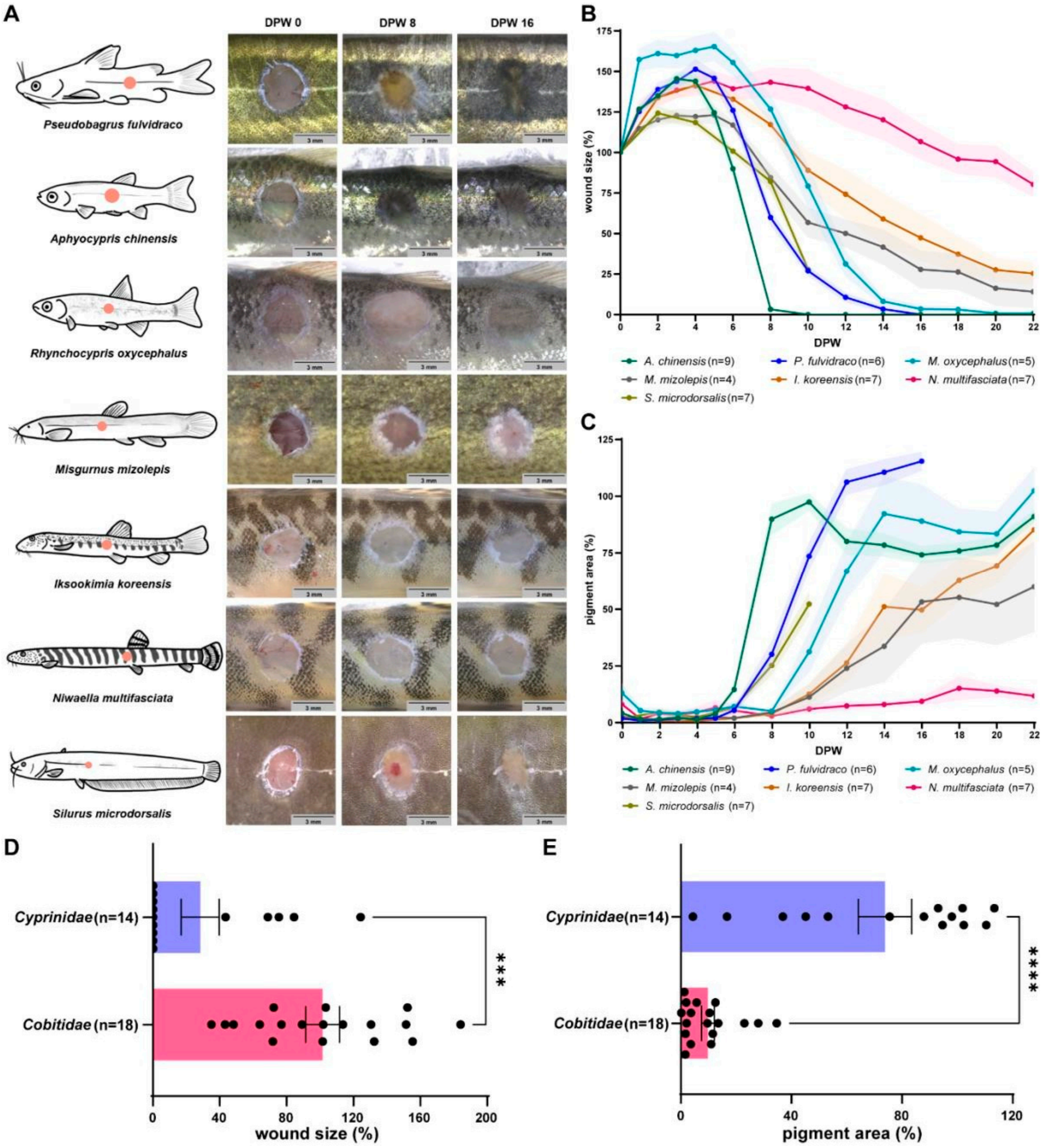

Figure S1. Wound healing rate of diverse fish species.

(A) Representative images of wounds. Wound images from 7 fish species at 0, 8, and 16 dpw. Scale bars: 3 mm. (B, C) Changes in wound size percent (B) and pigment percent (C). Wound size (pigment) percent: (wound size (pigment) at (n) dpw/0 dpw)*100. (D, E) Quantification of wound healing rate based on changes in wound size (D) and pigment (E). Wound healing rates between the families Cyprinidae and Cobitidae were compared by wound size (pigment) percent at 10 dpw. Sample size (n) is denoted next to the graph. *** p

≤ 0.001, **** p ≤ 0.0001.

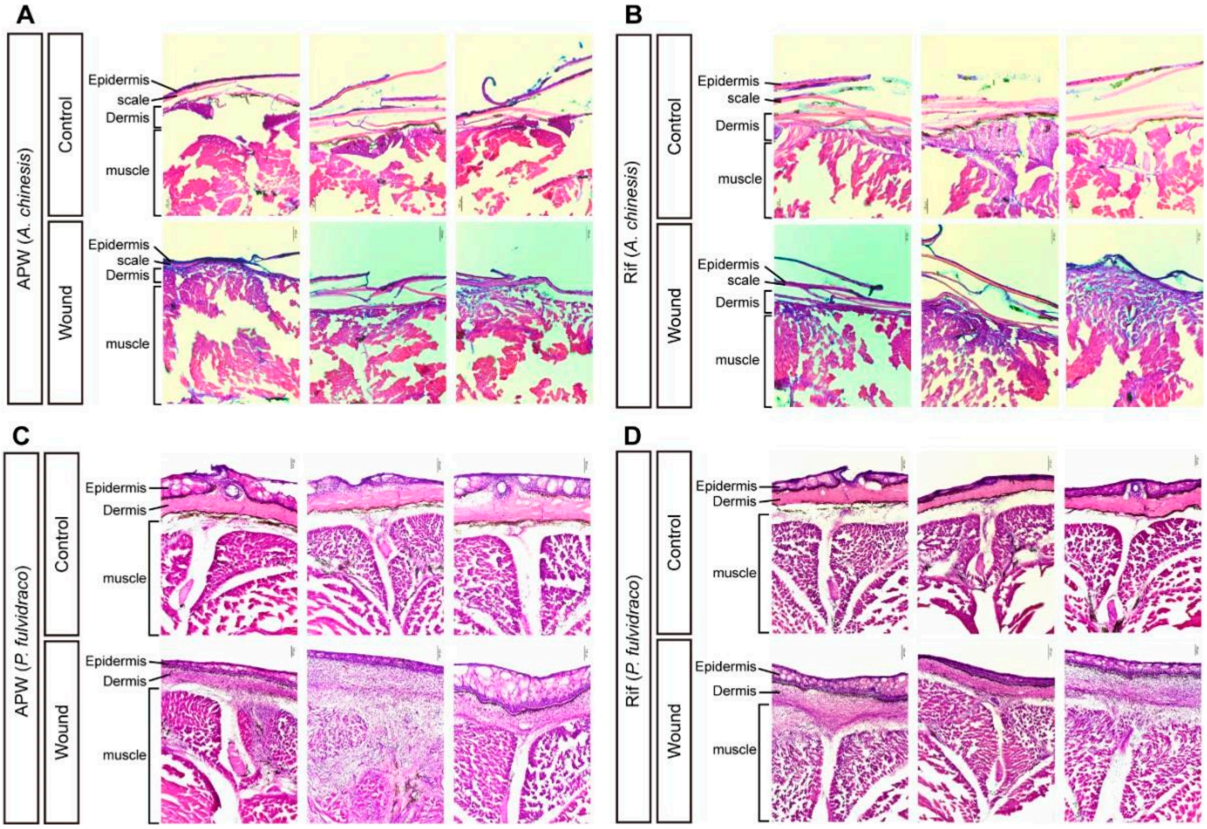

Figure S2. Skin organization after wound healing.
(A~D) H&E staining images showing transverse sections through the recovered wound tissue of Korean bullhead and Chinese bleak at 16 dpw. Representative images of 3 samples from each condition are shown here. (A, B) H&E staining on control side (top) and wounded side (bottom) of Chinese bleak reared in APW (A) and APW with rifampicin (B). (C, D) H&E staining on control side (top) and wounded side (bottom) of Korean bullhead reared in APW (C) and APW with rifampicin (D). Scale bars: 100 μm.

A

Aphyocypris chinensis APW	%
Muribaculaceae (Bacteroidia)	20.92
Muribaculaceae* (Bacteroidia)	11.71
Lactobacillus (Bacilli)	11.38
Muribaculum (Bacteroidia)	10.74
Lachnospiraceae* (Clostridia)	9.71
Campylobacter (Campylobacteria)	5.73
Bacteroides (Bacteroidia)	3.08
Bacteroidales** (Bacteroidia)	2.27
Oscillospiraceae* (Clostridia)	2.17
Akkermansia (Verrucomicrobiae)	2.11
Parasutterella (Gammaproteobacteria)	1.62
Lachnospiraceae_NK4A136_group (Clostridia)	1.39
Burkholderia-Caballeronia-Paraburkholderia (Gammaproteobacteria)	1.28
Desulfovibrio (Desulfovibrionia)	1.22
Clostridia_UCG-014 (Clostridia)	1.15
Oscillibacter (Clostridia)	0.84
Alistipes (Bacteroidia)	0.81
Colidextribacter (Clostridia)	0.66
Helicobacter (Campylobacteria)	0.61
Blautia (Clostridia)	0.52
others	10.09
Total Sum	100.00

Aphyocypris chinensis Rif	%
Muribaculaceae (Bacteroidia)	18.50
Lactobacillus (Bacilli)	11.12
Muribaculaceae* (Bacteroidia)	10.28
Muribaculum (Bacteroidia)	9.35
Lachnospiraceae* (Clostridia)	7.81
Campylobacter (Campylobacteria)	4.55
Bacteroides (Bacteroidia)	3.41
unclassified (Gammaproteobacteria)	2.96
Akkermansia (Verrucomicrobiae)	2.21
Bacteroidales** (Bacteroidia)	2.19
Oscillospiraceae* (Clostridia)	1.50
Clostridia_UCG-014 (Clostridia)	1.48
Parasutterella (Gammaproteobacteria)	1.44
Lachnospiraceae_NK4A136_group (Clostridia)	1.30
Enterococcus (Bacilli)	1.19
Bacillus (Bacilli)	1.00
Desulfovibrio (Desulfovibrionia)	1.00
Burkholderia-Caballeronia-Paraburkholderia (Gammaproteobacteria)	0.88
Oscillibacter (Clostridia)	0.74
Enhydrobacter (Gammaproteobacteria)	0.67
others	16.39
Total Sum	100.00

Pseudobagrus fulvidraco APW	%
Muribaculaceae (Bacteroidia)	17.75
Lactobacillus (Bacilli)	9.98
Muribaculaceae* (Bacteroidia)	9.78
Muribaculum (Bacteroidia)	8.68
Lachnospiraceae* (Clostridia)	7.55
Elizabethkingia (Bacteroidia)	4.61
Campylobacter (Campylobacteria)	4.24
Bacteroides (Bacteroidia)	2.77
unclassified (Gammaproteobacteria)	2.71
Niveispirillum (Alphaproteobacteria)	2.56
Akkermansia (Verrucomicrobiae)	2.28
Bacteroidales** (Bacteroidia)	2.16
unclassified (Verrucomicrobiae)	2.15
Oscillospiraceae* (Clostridia)	1.73
Parasutterella (Gammaproteobacteria)	1.40
Clostridia_UCG-014 (Clostridia)	1.33
Lachnospiraceae_NK4A136_group (Clostridia)	1.26
Desulfovibrio (Desulfovibrionia)	0.98
Comamonadaceae* (Gammaproteobacteria)	0.85
Burkholderia-Caballeronia-Paraburkholderia (Gammaproteobacteria)	0.82
others	14.42
Total Sum	100.00

Pseudobagrus fulvidraco Rif	%
Muribaculaceae (Bacteroidia)	19.09
Lactobacillus (Bacilli)	13.17
Muribaculaceae* (Bacteroidia)	11.08
Muribaculum (Bacteroidia)	10.12
Lachnospiraceae* (Clostridia)	9.08
Campylobacter (Campylobacteria)	4.46
Bacteroides (Bacteroidia)	3.76
Akkermansia (Verrucomicrobiae)	2.59
Bacteroidales** (Bacteroidia)	2.27
Parasutterella (Gammaproteobacteria)	1.82
Oscillospiraceae* (Clostridia)	1.59
Clostridia_UCG-014 (Clostridia)	1.57
Lachnospiraceae_NK4A136_group (Clostridia)	1.38
Burkholderia-Caballeronia-Paraburkholderia (Gammaproteobacteria)	1.09
unclassified (Gammaproteobacteria)	0.96
Enterobacterales** (Gammaproteobacteria)	0.95
Helicobacter (Campylobacteria)	0.89
Desulfovibrio (Desulfovibrionia)	0.80
Alistipes (Bacteroidia)	0.75
Oscillibacter (Clostridia)	0.69
others	11.89
Total Sum	100.00

B

Genus name	percentage	log2FoldChange	padj
***Aphyocypris chinensis* APW**			
Chryseobacterium (Bacteroidia)	0.35%	-6.13	8.08×10^{-4}
RF39 (Bacilli)	0.12%	-3.03	9.79×10^{-2}
ASF356 (Clostridia)	0.04%	-5.09	4.79×10^{-2}
Moraxellaceae* (Gammaproteobacteria)	0.04%	-5.05	5.23×10^{-2}
Comamonas (Gammaproteobacteria)	0.03%	-4.44	9.79×10^{-2}
***Aphyocypris chinensis* Rif**			
unclassified (Gammaproteobacteria)	2.96%	3.81	1.39×10^{-11}
Bacillus (Bacilli)	1.00%	6.02	5.95×10^{-2}
Cetobacterium (Fusobacteriia)	0.18%	6.90	8.58×10^{-3}
Sphingomonas (Alphaproteobacteria)	0.03%	4.76	6.49×10^{-2}
Intestinimonas (Clostridia)	0.03%	4.86	9.79×10^{-2}
***Pseudobagrus fulvidraco* APW**			
Elizabethkingia (Bacteroidia)	4.61%	-12.33	2.06×10^{-12}
Niveispirillum (Alphaproteobacteria)	2.56%	-11.16	8.62×10^{-15}
unclassified (Verrucomicrobiae)	2.15%	-11.00	1.46×10^{-14}
Shewanella (Gammaproteobacteria)	0.37%	-5.89	2.55×10^{-5}
Stella (Alphaproteobacteria)	0.37%	-8.43	7.00×10^{-8}
Fimbriimonadaceae* (Fimbriimonadia)	0.20%	-4.56	7.55×10^{-4}
Fluviicola (Bacteroidia)	0.14%	-7.02	1.13×10^{-5}
Arsenicibacter (Bacteroidia)	0.13%	-6.90	8.01×10^{-6}
Cloacibacterium (Bacteroidia)	0.12%	-6.93	4.93×10^{-4}
Crenobacter (Gammaproteobacteria)	0.11%	-6.66	1.13×10^{-5}
Enhydrobacter (Gammaproteobacteria)	0.11%	-4.77	1.97×10^{-2}
Cerasicoccus (Verrucomicrobiae)	0.06%	-5.84	6.32×10^{-4}
OM190 (Planctomycetota***)	0.06%	-3.44	8.98×10^{-2}
Sphingopyxis (Alphaproteobacteria)	0.03%	-5.14	2.49×10^{-3}
pirellula (Planctomycetes)	0.03%	-5.01	3.98×10^{-3}
unclassified (Alphaproteobacteria)	0.03%	-4.80	2.19×10^{-2}
Legionella (Gammaproteobacteria)	0.02%	-4.24	4.89×10^{-2}
Mycobacterium (Actinobacteria)	0.02%	-4.19	7.09×10^{-2}
Proteus (Gammaproteobacteria)	0.02%	-4.56	5.46×10^{-2}
Candidatus Odyssella (Alphaproteobacteria)	0.02%	-3.96	8.98×10^{-2}
Candidatus Branchiomonas (Gammaproteobacteria)	0.02%	-4.14	7.17×10^{-2}
Candidatus similichlamydia (Chlamydiae)	0.02%	-4.19	5.57×10^{-2}
methylotenera (Gammaproteobacteria)	0.02%	-4.62	2.85×10^{-2}
Noviherbaspirillum (Gammaproteobacteria)	0.02%	-4.19	2.85×10^{-2}
CCM19a (Gammaproteobacteria)	0.02%	-4.29	6.54×10^{-2}
intestinimonas (Clostridia)	0.02%	-4.08	5.57×10^{-2}
Dyadobacter (Bacteroidia)	0.02%	-4.52	3.57×10^{-2}
Weeksellaceae* (Bacteroidia)	0.02%	-4.29	6.54×10^{-2}
Geothermomicrobium (Bacilli)	0.02%	-4.16	7.17×10^{-2}
Planococcaceae* (Bacilli)	0.02%	-4.32	6.54×10^{-2}
Vogesella (Gammaproteobacteria)	0.02%	-4.29	6.54×10^{-2}
Fusobacterium (Fusobacteriia)	0.01%	-3.84	7.04×10^{-2}
Empedobacter (Bacteroidia)	0.01%	-3.69	6.35×10^{-2}
Nitrosomonas (Gammaproteobacteria)	0.01%	-3.90	9.67×10^{-2}
***Pseudobagrus fulvidraco* Rif**			
Pseudoxanthomonas (Gammaproteobacteria)	0.12%	3.54	0.05
Chitinophagaceae* (Bacteroidia)	0.06%	3.75	0.03
Enterococcus (Bacilli)	0.04%	3.84	0.05
unclassified (Firmicutes***)	0.04%	5.01	0.02
Enterococcus (Bacilli)	0.04%	3.84	0.05
UCG-003 (Clostridia)	0.03%	4.57	0.03
Barnesiellaceae* (Bacteroidia)	0.03%	4.62	0.05
Faecalibacterium (Clostridia)	0.02%	4.39	0.04
Gordonibacter (Coriobacteriia)	0.02%	3.88	0.07
UC5-1-2E3 (Clostridia)	0.02%	3.78	0.07
Bosea (Alphaproteobacteria)	0.01%	3.78	0.06

Figure S3. Microbiome abundance in each group.

(A) The top 20 taxonomic diversity and relative abundance in Figure 3. Genus (class) is shown and if the genus level is not identifiable, an additional level is included. * family, **order. (B) Relative abundance of bacteria shown in Figure 4 at the genus (class) level.
